# Understanding controversies in the α-ω and ω-β phase transformations of zirconium from nonhydrostatic thermodynamics

**DOI:** 10.1038/s41598-019-53088-3

**Published:** 2019-11-15

**Authors:** Lin Zhang, Ying-Hua Li, Yan-Qin Gu, Ling-Cang Cai

**Affiliations:** 1 National Key Laboratory of Shock Wave and Detonation Physics, Institute of Fluid Physics, CAEP, P.O. Box 919-102, Mianyang, 621900 China; 2 Center for Compression Science, CAEP, P.O. Box 919-150, Mianyang, 621900 China

**Keywords:** Phase transitions and critical phenomena, Thermodynamics

## Abstract

Significant debate has been noted in the α-ω and ω-β phase transformations of zirconium. The initial pressure of the α-to-ω transformation at room temperature has been reported to vary from 0.25 to 7.0 GPa, while the hydrostatic transformation is believed to occur at approximately 2.2 GPa. Shear stress is commonly considered as a key factor leading to the discrepancy. However, the principal mechanisms previously proposed concluded that the phase transformation pressure would be decreased in the presence of shear stress. The experimental results of the α-ω transformation in zirconium are contrary to this conclusion. In the ω-β phase diagram of zirconium, the d*T*/d*P* along the phase boundary near the α-ω-β triple-point was reported to be either positive or negative, but no theoretical explanation, especially a quantitative one, has been proposed. This article aimed to quantitatively investigate and explain the controversies reported in the α-ω and ω-β phase transformations of zirconium by applying a new nonhydrostatic thermodynamic formalism for solid medium, which has recently been proposed and is capable of quantitatively estimating the impact of shear stress on phase transformations in solids.

## Introduction

The phase structure, phase transformation and phase diagram of zirconium have attracted the interest of many researchers because of their scientific significance and widespread applications in aerospace, nuclear, and biomedical industries^1–21^; however, studies in the past few decades have revealed some controversies in its α-ω and ω-β phase transformations. For the α-ω transformation, the initial transition pressure at room temperature was found to vary from 0.25 to 7.0 GPa^1–5,11,12^, which exhibits a great discrepancy. For the ω-β phase transformation, earlier measurements discovered a positive *dT*/*dP* slope of approximately 6 K/GPa along the ω-β phase boundary near the triple point of the α-ω-β phase diagram^22^. It was found that the ω phase can be transformed into the β phase at room temperature by pressure at approximately 30 ± 2, 33 and 35 ± 3 GPa^6–8^, as well as by shock at approximately 26 GPa^7,23^. These indicate that the ω-β phase boundary of zirconium must return toward the room temperature axis at high pressure at some point. However, thus far, no published reports of theoretical multiphase equation of state (mEOS) have noted such behaviour. Zhang *et al*.^9,10^ re-measured the phase diagram of zirconium using synchrotron x-ray diffraction and time-of-flight neutron scattering techniques, and a negative *dT*/*dP* slope of approximately 15.5 K/GPa was obtained along the ω-β phase boundary near the triple point, which was contrary to the earlier results. Similar controversies were also found in many other substances^13,19,24–27^. Although some studies were devoted to explain these controversies^20,21^, their understanding remains an open issue. Impurity is often considered as a main factor that may cause these controversies. In general, impurity may change the energy barrier for phase transformation, resulting in a change in its transition pressure^28^. Shear stress is another factor exerting high impact on phase transformation. There are two principal viewpoints on how shear stress affects phase equilibrium and phase transformation. One states that shear stress is influential to the potential energy for phase transformation, however it is commonly accepted that shear stress usually decreases the height of the potential barrier^29–34^. The other viewpoint^20,21,35–39^ explains that shear stress causes plastic deformation of the sample and leads to dislocations in return, and the dislocations are further densely piled up against grain boundary or other obstacles, thus strong concentrators of stress tensor are generated. As a result, the local stresses near stress concentrators may reach the required level for high-pressure phase nucleation, even though the externally applied pressure is much lower than the transition pressure. Two review papers focused on the studies can be found in^40,41^. These two viewpoints concluded that shear stress leads to a decrease in transition pressure. Although the conclusion was supported by many experiments performed on some substances, the α-ω transformation in zirconium is contrary to this because the hydrostatic transition pressure at room temperature of the transformation is usually believed to be approximately 2.2 GPa^4–7^, much lower than most experimental measured values. Thus, a more reasonable and persuasive theoretical interpretation is highly necessary. In a recent study, we proposed a nonhydrostatic thermodynamic formalism^42^ (referred to as ZLC formalism hereinafter), which is capable of quantitatively estimating the impact of shear stress on phase transformations in a solid. According to the formalism, the transition pressure of a solid-solid phase transformation is extremely sensitive to the shear stress level and the material shear modulus as a function of pressure and temperature of both relevant phases, i.e., the *G*(*P*, *T*) relationships for both phases. This conclusion gives birth to a new mechanism of the influence of impurity on phase transformation. In other words, by changing the yield stress level and the *G*(*P*, *T*) relationship of the material, a tiny amount of impurities may exert great impact on phase transformations. In this article, we aimed to explain the controversies observed in the α-ω and ω-β transformations in zirconium by applying the formalism. We believe that a comprehensive quantitative explanation of these controversies may provide a general understanding and a theoretical approach to explain similar controversies that were widely observed in pressure-induced phase transformations in other solids.

## Methods

### ZLC formalism

ZLC formalism is an approximate approach for nonhydrostatic thermodynamics which is applicable for phase equilibrium, phase diagram and phase transformation problems under nonhydrostatic situations from a macroscopic view, and is based on the following assumptions that are widely adopted in solid mechanics:

1. The object system could be regarded as a homogenous continuum, and the heterogeneities in meso- and micro-scales could be ignored or managed based on the average.
2. The substance of two phases that coexist at equilibrium could be regarded as a solution (completely mixed together), and the effect of interface between the two phases might be ignored.
3. A quantity *E*_d_ was introduced to depict the total stored energy produced by various meso- and micro-scales defects such as dislocations and grain boundaries. *E*_d_ could be dealt with as being homogenously stored throughout the object, based on the average. In addition, the inelastic work done by internal stresses should be the dominant contribution to the increment of *E*_d_.
4. The constitutive relationships among the components of deviatoric stress and deviatoric elastic strain are linear at fixed pressure and temperature. However, the elastic coefficients may change with pressure and temperature.

Based on the above assumptions, the first law of thermodynamics under nonhydrostatic situation was derived as follows:

1 $$d{E}_{v}={T}_{{\rm{eff}}}dS-{P}_{{\rm{eff}}}dV+\mu dn$$

in which:

2 $${E}_{v}=E-{E}_{d}-{E}_{\tau }$$

3 $${T}_{{\rm{eff}}}=T-\mathop{\sum }\limits_{{\rm{i}},\,{\rm{j}},\,{\rm{m}},\,{\rm{n}}=1}^{3}\,\frac{1}{2}v\,[{(\frac{\partial {C}_{{\rm{ijmn}}}}{\partial T})}_{P}+{(\frac{\partial {C}_{{\rm{ijmn}}}}{\partial P})}_{T}{(\frac{\partial P}{\partial T})}_{v}]{(\frac{\partial T}{\partial s})}_{v}{\xi }_{{\rm{ij}}}^{e}{\xi }_{{\rm{mn}}}^{e}$$

4 $$\begin{array}{rcl}{P}_{{\rm{eff}}} & = & P+\mathop{\sum }\limits_{{\rm{i}},{\rm{j}}=1}^{3}\,\frac{1}{2}{\tau }_{{\rm{ij}}}{\xi }_{{\rm{ij}}}^{e}+\mathop{\sum }\limits_{{\rm{i}},{\rm{j}},{\rm{m}},{\rm{n}}=1}^{3}\frac{1}{2}v\,[{(\frac{\partial {C}_{{\rm{ijmn}}}}{\partial P})}_{T}{(\frac{\partial P}{\partial v})}_{T}+{(\frac{\partial {C}_{{\rm{ijmn}}}}{\partial T})}_{P}{(\frac{\partial T}{\partial v})}_{s}\\  &  & +{(\frac{\partial {C}_{{\rm{ijmn}}}}{\partial P})}_{T}{(\frac{\partial P}{\partial T})}_{v}{(\frac{\partial T}{\partial v})}_{s}]{\xi }_{{\rm{ij}}}^{e}{\xi }_{{\rm{mn}}}^{e}\end{array}$$

where *P*, *T*, *V*, *S*, *E* and *E*_τ_ are the pressure, temperature, volume, entropy, total energy and total elastic potential energy related to shear deformation of the system, respectively; *μ* is the general chemical potential, *n* is the number of moles of the substance; *C*_ijmn_(i, j, m, n = 1–3) are the elastic coefficients, components of a fourth-order tensor, and satisfy *C*_ijmn_ = *C*_mnij_; *τ*_ij_ and ${\xi }_{{\rm{ij}}}^{e}$ (ij = 1–3) are the components of deviatoric stress and deviatoric elastic strain, respectively; lowercase “*s*” and “*v*” denote the specific entropy and volume per mass unit; *T*_eff_ and *P*_eff_ are the conjugate quantities to entropy and volume with respect to the thermodynamic potential *E*_V_, and have the units of temperature and pressure, respectively. It is worth noting that *T*_eff_ and *P*_eff_ are derived to correlate with the constitutive relationships between stress and strain, and this has essential significance; this implies that deviatoric quantities can effectively affect volumetric quantities in non-hydrostatic situations, while in classical solid mechanics volumetric quantities are usually believed to have no relationship with deviatoric quantities. By the variation method, the two-phase equilibrium conditions under nonhydrostatic situation were derived as follows:

5 $${{\rm{T}}}^{{\rm{{\rm I}}}}={T}^{{\rm{{\rm I}}}{\rm{{\rm I}}}},\,{P}^{{\rm{{\rm I}}}}={P}^{{\rm{{\rm I}}}{\rm{{\rm I}}}},\,{\tau }_{{\rm{ij}}}^{{\rm{{\rm I}}}}={\tau }_{{\rm{ij}}}^{{\rm{{\rm I}}}{\rm{{\rm I}}}},\,{\mu }^{{\rm{{\rm I}}}}={\mu }^{{\rm{{\rm I}}}{\rm{{\rm I}}}}$$

where the superscripts “I” and “II” represent variables for phase I and II, respectively. Furthermore, the expression for the general chemical potential was derived as:

6 $$\mu =({E}_{V}-{T}_{{\rm{eff}}}S+{P}_{{\rm{eff}}}V)/n={e}_{V}-{T}_{{\rm{eff}}}s+{P}_{{\rm{eff}}}v$$

For isotropic solids (in the strict sense, when an isotropic solid is subjected to nonhydrostatic load, its isotropic symmetry will be broken due to the nonhydrostatic stress produced inside the body; however, for most engineering materials, especially metals, the isotropic approximation for stressed bodies is extensively adopted, especially in engineering), the relationships between deviatoric stresses and deviatoric strains become ${\tau }_{{\rm{ij}}}=2G{\xi }_{{\rm{ij}}}^{e}$ (i, j = 1–3), in which *G* = *G*(*P*, *T*) is the material shear modulus, and the expressions for the quantities *T*_eff_ and *P*_eff_ are changed to

7 $${T}_{{\rm{eff}}}=T-\frac{{e}_{\tau }}{G}[{(\frac{\partial G}{\partial P})}_{T}{(\frac{\partial P}{\partial T})}_{v}+{(\frac{\partial G}{\partial T})}_{P}]{(\frac{\partial T}{\partial s})}_{v}$$

8 $${P}_{{\rm{eff}}}=P+\frac{1}{v}{e}_{\tau }+\frac{{e}_{\tau }}{G}{(\frac{\partial G}{\partial P})}_{T}{(\frac{\partial P}{\partial v})}_{T}+\frac{{e}_{\tau }}{G}[{(\frac{\partial G}{\partial T})}_{P}+{(\frac{\partial G}{\partial P})}_{T}{(\frac{\partial P}{\partial T})}_{v}]{(\frac{\partial T}{\partial v})}_{s}$$

where *e*_*τ*_ is the specific elastic shear deformation energy, which is defined as follows:

9 $${e}_{\tau }=(1/2)v\cdot \mathop{\sum }\limits_{{\rm{i}},\,{\rm{j}}=1}^{3}\,{\tau }_{{\rm{ij}}}{\xi }_{{\rm{ij}}}^{e}=v\cdot {J}_{2}/(2G)$$

*J*_2_ refers to the second invariant of the deviatoric stress tensor. Making use of Eqs (5–9), the generalised Clausius-Clapyron relationship for isotropic solids under the above restrictions is derived as follows:

10 $$\begin{array}{c}\{[{s}^{{\rm{I}}}{(\frac{\partial {T}_{{\rm{eff}}}^{{\rm{I}}}}{\partial P})}_{{\rm{T}},{{\rm{J}}}_{2}}-{s}^{{\rm{II}}}{(\frac{\partial {T}_{{\rm{eff}}}^{{\rm{II}}}}{\partial P})}_{{\rm{T}},{{\rm{J}}}_{2}}]-[{v}^{{\rm{I}}}{(\frac{\partial {P}_{{\rm{eff}}}^{{\rm{I}}}}{\partial P})}_{{\rm{T}},{{\rm{J}}}_{2}}-{v}^{{\rm{II}}}{(\frac{\partial {P}_{{\rm{eff}}}^{{\rm{II}}}}{\partial P})}_{{\rm{T}},{{\rm{J}}}_{2}}]\}dP\\ +\{[{s}^{{\rm{I}}}{(\frac{\partial {T}_{{\rm{eff}}}^{{\rm{I}}}}{\partial T})}_{{\rm{P}},{{\rm{J}}}_{2}}-{s}^{{\rm{II}}}{(\frac{\partial {T}_{{\rm{eff}}}^{{\rm{II}}}}{\partial T})}_{{\rm{P}},{{\rm{J}}}_{2}}]-[{v}^{{\rm{I}}}{(\frac{\partial {P}_{{\rm{eff}}}^{{\rm{I}}}}{\partial T})}_{{\rm{P}},{{\rm{J}}}_{2}}-{v}^{{\rm{II}}}{(\frac{\partial {P}_{{\rm{eff}}}^{{\rm{II}}}}{\partial T})}_{{\rm{P}},{{\rm{J}}}_{2}}]\}dT\\ +\{[{s}^{{\rm{I}}}{(\frac{\partial {T}_{{\rm{eff}}}^{{\rm{I}}}}{\partial {J}_{2}})}_{{\rm{P}},{\rm{T}}}-{s}^{{\rm{II}}}{(\frac{\partial {T}_{{\rm{eff}}}^{{\rm{II}}}}{\partial {J}_{2}})}_{{\rm{P}},{\rm{T}}}]-[{v}^{{\rm{I}}}{(\frac{\partial {P}_{{\rm{eff}}}^{{\rm{I}}}}{\partial {J}_{2}})}_{{\rm{P}},{\rm{T}}}-{v}^{{\rm{II}}}{(\frac{\partial {P}_{{\rm{eff}}}^{{\rm{II}}}}{\partial {J}_{2}})}_{{\rm{P}},{\rm{T}}}]\}d{J}_{2}\end{array}=0$$

The expressions for the partial differentiations in Eq. (10) can be found in our previous work^42^.

ZLC formalism indicates that for a solid in nonhydrostatic stressed states, the traditional phase boundary line between two solid phases in (*P*, *T*) space is extended to a phase boundary face in (*P*, *T*, *J*_2_) space under the isotropic approximation, and the *P* = *P*(*T*, *J*_2_) phase diagram can be constructed from the *P* = *P*(*T*) phase diagram using Eq. (10).

### mEOS for zirconium

**Figure 1:**
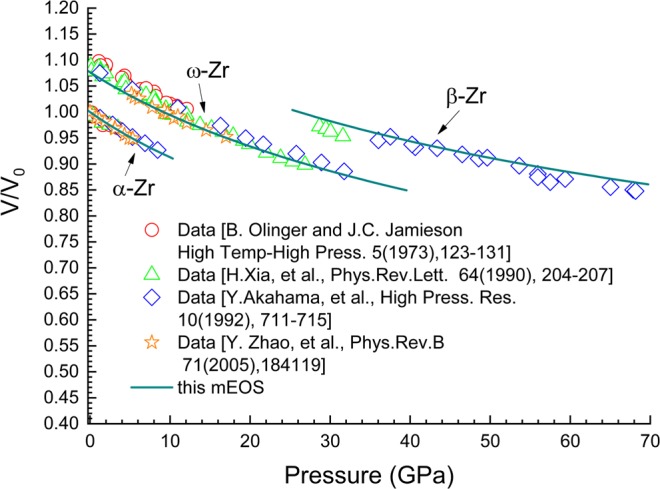
Isothermal line for α-,ω- and β-Zr at room temperature. Scatter symbols are experimental data from^2,6,8,51^; solid line represents theoretical results. Volume is normalised to the value at ambient conditions *V*_0_. A value of 0.1 was added to V/V_0_ for ω-Zr and 0.2 to that for β-Zr in order to make a clear separation.

**Figure 2:**
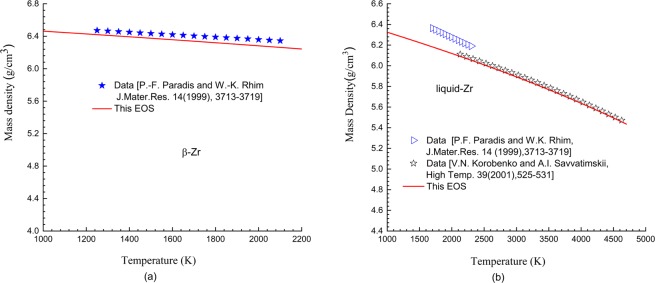
Isobaric lines under ambient pressure for β- and liquid-Zr. Scatter symbols are experimental data^52,53^; solid lines represent theoretical results.

**Figure 3:**
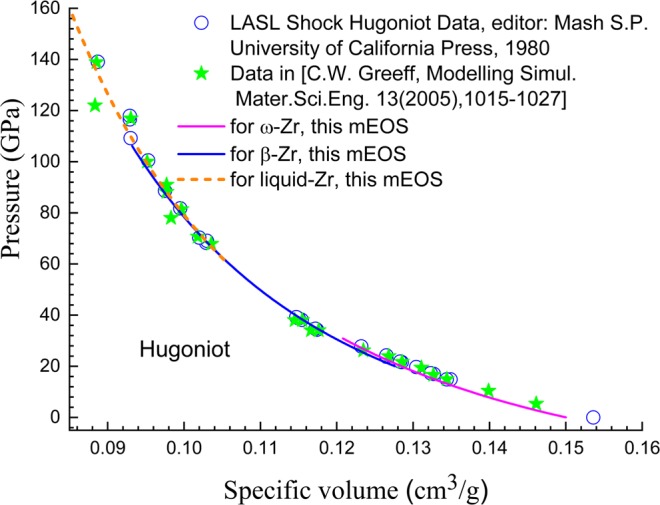
Hugoniot lines of zirconium. Scatter symbols are experimental data^54,55^; lines represent theoretical results.

To quantitatively evaluate the shear stress effects on phase transformation through ZLC formalism, the hydrostatic multiphase equation of states and the *G* = *G*(*P*, *T*) relationships for related phases of the material must be constructed. The mEOS of α-, ω-, β- and liquid-Zr have been constructed in this article. The liquid state was included because the *G*(*P*, *T*) model we adopted in this work correlates with the melt line, i.e., the phase boundary between the corresponding solid and liquid phases. The mEOS were constructed using an average mean field potential model which is based on calculations of the specific Helmholtz free energy *f*(*v*, *T*). The equations used to calculate *f*(*v*, *T*) in this work are the same as those in our previous work^42^, which are summarised as follows:

11 $$f(v,T)={e}_{c}(v)+{f}_{{\rm{ion}}}(v,T)+{f}_{{\rm{el}}}(v,T)$$

12 $${f}_{{\rm{ion}}}(v,T)=-\,N{k}_{B}T(\frac{3}{2}\,\mathrm{ln}\,\frac{m{k}_{B}T}{2\pi {\hslash }^{2}}+\,\mathrm{ln}\,{v}_{f})$$

13 $${v}_{f}=4\pi {\int }_{0}^{R}\,{r}^{2}\,\exp \,[-\frac{g(r,v)}{{k}_{B}T}]dr$$

14 $$g(r,v)={g}_{0}(v)\cdot h(r,v)$$

15 $${g}_{0}(v)=\frac{m{f}_{0}^{2}}{h^{\prime\prime} (0,v)}\frac{q^{\prime} (v)}{q^{\prime} ({v}_{0})}{(\frac{v}{{v}_{0}})}^{4/3-\alpha }$$

16 $$q(v)={P}_{c}(v){v}^{\alpha }$$

17 $$h(r,v)=\frac{1}{{(R+r)}^{n}}+\frac{1}{{(R-r)}^{n}}-\frac{2}{{R}^{n}}$$

18 $${P}_{c}(v)=3{B}_{0}\frac{1-x}{{x}^{2}}\exp [\eta (1-x)];\,x={(v/{v}_{0})}^{1/3};\,\eta =3({B^{\prime} }_{0}-1)/2$$

19 $${e}_{c}(v)={e}_{0}+\frac{4{B}_{0}{v}_{0}}{{({B^{\prime} }_{0}-1)}^{2}}\{1-[1-\eta (1-x)]{e}^{\eta (1-x)}\}$$

20 $${f}_{{\rm{el}}}(v,T)=-\,\varGamma {(\frac{v}{{v}_{r}})}^{\delta }{T}^{2}$$

where *e*_c_ is the static energy at zero temperature, *f*_ion_ is the free energy of ion motion and *f*_el_ is the free energy due to the thermal excitation of electrons. The details of derivation of the above equations and the significance of the parameters please refer to our previous article^42^. The final EOS parameters for α-, ω-, β- and liquid-Zr are listed in Table 1, in which the parameter *Γ* was determined from the low-temperature heat capability data^43^, others were fitted to various experimental measurements of thermodynamic and phase transformation properties. Figures 1–3 as well as Tables 2 and 3 present various comparisons between theoretic predictions and experimental results, which demonstrate that the mEOS constructed for zirconium in this work have high precision.

**Table 1:** EOS parameters for α, ω, β and liquid-zirconium.

	*B*_0_ (GPa)	*B*′	*V*_0_ (m^3^/kg)	*f*_0_ (s^−1^)	*e*_0_ (J/kg)	*α*	*n*_0_	*Γ* (J/kg.K^2^)	v_r_ (m^3^/kg)	*δ*
α	94	4.0	1.527e-4	2.0e13	0	4/3	0.5	0.0153	1.527e-4	0.7
ω	100	3.2	1.4945e-4	2.162e13	−38.585548	4/3	0.5	0.0153	1.527e-4	0.7
β	79	3.9	1.52e-4	1.75e13	40438.613	4/3	0.5	0.0153	1.527e-4	0.7
L	77	4.1	1.535e-4	9.0e12	4086.7004	4/3	0.5	0.0153	1.527e-4	0.7

**Table 2:** Specific volumes of α-Zr at various high-pressure and -temperature points.

*P* (GPa)	*T* (°K)	*V*/*V*_0_ (expt)	*V*/*V*_0_ (theoretic)	(theoretic-expt)/expt
1.01	473	0.99184	0.99315	0.13%
2.08	474	0.98175	0.98221	0.05%
3.36	474	0.96908	0.96983	0.08%
1.59	673	0.99055	0.99158	0.1%
1.27	674	0.99356	0.99498	0.14%
2.33	674	0.98218	0.98401	0.19%
3.63	674	0.96888	0.97135	0.26%
1.45	873	0.99678	0.99768	0.09%
2.63	873	0.98218	0.98535	0.32%
3.91	874	0.96951	0.97283	0.34%
2.67	918	0.98497	0.98594	0.1%

Experimental values are cited from Zhao *et al*.^51^.

**Table 3:** Specific volumes of β-Zr at various high-pressure and -temperature points.

*P* (GPa)	*T* (°K)	*V*/*V*_0_ (expt)	*V*/*V*_0_ (theoretic)	(theoretic-expt)/expt
0	973	1.02491	1.0154	−0.93%
6.38	973	0.94159	0.94044	−0.12%
7.37	972	0.93515	0.93094	−0.45%
8.63	898	0.91604	0.91809	0.22%
8.63	973	0.91991	0.91951	−0.04%
10.54	974	0.90337	0.90331	−0.01%
13.4	975	0.88082	0.88127	0.05%
15.38	873	0.85935	0.8658	0.75%
14.82	930	0.86343	0.87048	0.82%
14.49	973	0.87116	0.87343	0.26%

Experimental values are cited from Zhao *et al*.^51^.

**Figure 4:**
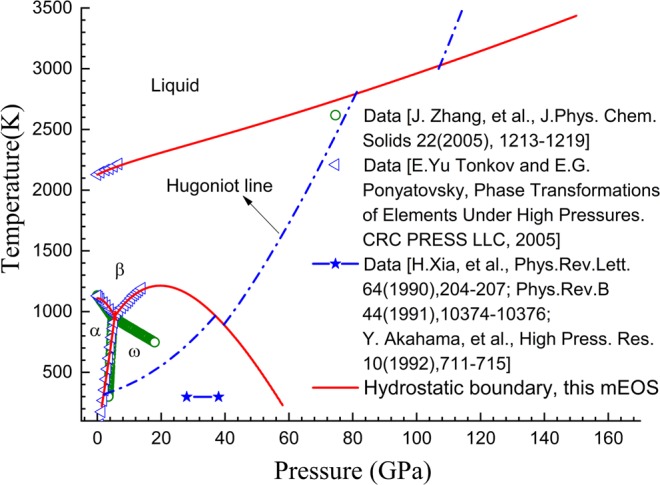
Hydrostatic phase diagram of zirconium. Scatter symbols are experimental data^6–9,22^; lines represent theoretical results.

Figure 4 presents the hydrostatic phase diagram of α, ω, β and liquid phases. It shows that the theoretical boundaries agree well with the experimental data that were collected by Tonkov and Ponyatovsky^22^. Moreover, the α-to-ω transformation is predicted to occur at approximately 1.85 GPa under room temperature, accompanying a volume change of (v_α_ − v_ω_)/v_ω_ = 2.3%, which coincides with the experimental results^22^. The theoretical slope d*T*/d*P* of the ω-β phase boundary was calculated to be positive near the α-ω-β triple point; however, as pressure increases, it gradually becomes negative. Therefore, the theoretical ω-β phase boundary finally returns toward the room temperature axis. This explains the occurrence of the pressure-induced ω-to-β phase transformation at room temperature observed in some experiments. Here, we would like to highlight our mEOS for the curvature of the theoretical ω-β phase boundary of zirconium. We found no other published mEOS of zirconium having such curvature property. Through our mEOS, the ω-to-β transformation is predicted to take place at approximately 56.0 GPa under hydrostatic loading at room temperature, and at approximately 36.8 GPa under shock, whereas the corresponding experimental results are approximately (30 ± 2)–(35 ± 3) GPa^6–8^ and 26.0 GPa^7,23^, respectively. These discrepancies can be explained by the shear stress impacts, which will be reported later in this article.

### *G*(*P*, *T*) relationships

Numerous studies have focused on the variation of shear modulus with pressure and temperature for solid materials, on which we made a brief review in our previous article^42^. In this article, we use the following equation:

21 $$G(P,T)=\frac{3[1-2\sigma (P,T)]}{2[1+\sigma (P,T)]}B(P,T)$$

where *B* is the bulk modulus, and its relationship of *B*(*P*, *T*) can be calculated from EOS; σ is the Poisson ratio. There are some advantages to calculating *G*(*P*, *T*) through the equation, because it is well known that the Poisson ratio varies only slightly with pressure and temperature. Many experimental measurements on various materials have indicated that the σ(*T*) relationship under constant pressure can be reasonably approximated by linear line, while the σ(*P*) relationship under constant temperature deviates slightly from linear. Thus, we experimentally write:

22 $$\sigma (P,T)={\sigma }_{r}(P,{T}_{r})+\frac{{\sigma }_{c}(P,{T}_{c}(P))-{\sigma }_{r}(P,{T}_{r})}{{T}_{c}(P)-{T}_{r}}(T-{T}_{r})$$

where *T*_r_ is a reference temperature, σ_r_(*P*, *T*_r_) the pressure-dependent Poisson ratio at the reference temperature and *T*_c_(*P*) the pressure-dependent critical temperature at which the Poisson ratio begins to rapidly increase to 0.5 as the temperature increases close to the melting point. σ_c_(*P*, *T*_c_(*P*)) is the critical Poisson ratio corresponding to the critical point (*P*, *T*_c_(*P*)). For σ_c_(*P*, *T*_c_(*P*)) and σ_r_(*P*, *T*_r_), we proposed the following expressions^42^:

23 $${\sigma }_{c}(P,{T}_{c}(P))={\sigma }_{c}(0,{T}_{c}(0))+{\sigma }_{c}^{1}\,\exp \,[\,-\,({T}_{m}-{T}_{c})/{T}_{0}]$$

24 $${\sigma }_{r}(P,{T}_{r})={\sigma }_{r}^{0}+{\sigma }_{r}^{1}\,\exp \,({\sigma }_{r}^{2}P)$$

25 $${T}_{c}(P)=\alpha \cdot {T}_{m}(P)$$

where σ_r_^0^, σ_r_^1^, σ_r_^2^, σ_c_(0, Tc(0)), σ_c_^1^, *T*_0_ and *α* are constant parameters.

There are some data on the relationship between the Poisson ratio, pressure, and temperature for zirconium. Fisher *et al*.^44^ determined the pressure derivatives of the single-crystal elastic moduli of α phase experimentally, thus providing some data on the σ(*P*) relationship, and these data can be well fitted by Eq. (24) with the parameters (σ_r_^0^, σ_r_^1^, σ_r_^2^) being (0.41915, −0.08792, −0.07034 GPa^−1^). Lu *et al*.^45^ reported an experimental value of 0.29 for ω phase at ambient conditions. Recently, Liu *et al*.^46^ measured the values for α and ω phases at ambient conditions, being 0.331 and 0.311 respectively. Some high-pressure experimental values for ω phase at room temperature were reported in another of their ref.^47^, and these data are well fitted by Eq. (24) with the parameters (σ_r_^0^, σ_r_^1^, σ_r_^2^) being (0.3392, −0.04921, −0.18431 GPa^−1^). In theory, Zhang *et al*.^48^ calculated by first-principles the variation of Poisson ratio with pressure for α, ω and bcc phases at absolute zero, which might be fitted by Eq. (24) with the parameters (σ_r_^0^, σ_r_^1^, σ_r_^2^) being (1.28976, −0.96602, −0.0042 GPa^−1^) for α phase, (0.4885, −0.19503, −0.01902 GPa^−1^) for ω phase, and (0.39435, 0.10115, −0.05739 GPa^−1^) for bcc phase, respectively. Wang *et al*.^49^ also calculated the values for bcc phase under various high pressures, which might be fitted by Eq. (24) with the parameters (σ_r_^0^, σ_r_^1^, σ_r_^2^) being (0.32361, 0.13328, −0.03152 GPa^−1^).

## Results and Discussion

**Figure 5:**
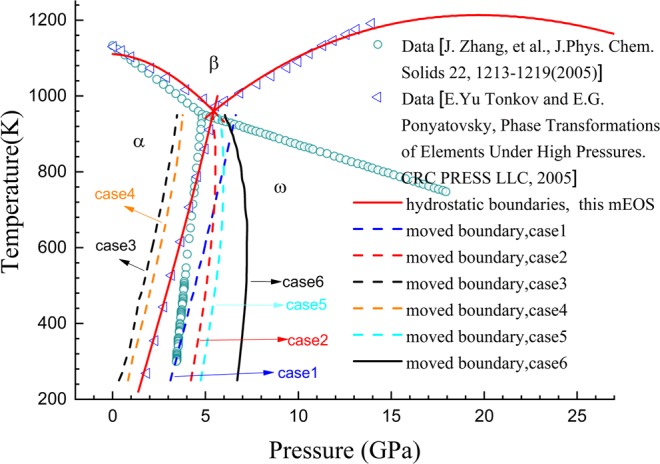
Changes of the α-ω phase boundary of zirconium that are caused by shear stresses. The results are examined in the situations listed in Table 4.

For the α-ω transformation, we examined the movement of the phase boundary in (*P*, *T*) space at some fixed shear stress levels. Six situations in total have been calculated. The details of *σ*(*P*, *T*) relationship for both α and ω phases, as well as the *J*_2_ levels for each situation are presented in Table 4, the altered *P*-*T* phase boundaries are shown in Fig. 5. In Table 4, the altered transition pressures at room temperature (assumed to be 293 K) under the six situations are also listed.

**Table 4:** The relationships of *σ*(*P*, *T*) that were used for α and ω phases when calculating the change of the α-ω phase boundary caused by shear stresses.

	α-Zr		ω-Zr		Shear stress *J*_2_(GPa^2^)	Transition pressure at 293 K
	*ν*_r_(*P*, *T*_r_)	*ν*_c_(*P*, *T*_c_)	*ν*_r_(*P*, *T*_r_)	*ν*_c_(*P*, *T*_c_)		
case 1	constant value 0.33; *T*_r_ = 293 K	constant value 0.48; *T*_c_ = 0.95*T*_m_	constant value 0.29; *T*_r_ = 293 K	Constant value 0.48; *T*_c_ = 0.95*T*_m_	1.0	3.36 GPa
case 2	constant value 0.33; *T*_r_ = 293 K.	constant value 0.48; *T*_c_ = 0.95*T*_m_	fitted to^47^; *T*_r_ = 293 K	Constant value 0.48; *T*_c_ = 0.95*T*_m_	1.0	4.38 GPa
case 3	fitted to^44^; *T*_r_ = 293 K	constant value 0.48; *T*_c_ = 0.95*T*_m_	fitted to^47^; *T*_r_ = 293 K	Constant value 0.48; *T*_c_ = 0.95*T*_m_	1.0	0.64 GPa
case 4	fitted to^44^; *T*_r_ = 293 K	constant value 0.48; *T*_c_ = 0.95*T*_m_	fitted to^48^; *T*_r_ = 0 K	Constant value 0.48; *T*_c_ = 0.95*T*_m_	1.0	1.02 GPa
case 5	constant value 0.33; *T*_r_ = 293 K	constant value 0.48; *T*_c_ = 0.95*T*_m_	fitted to^48^; *T*_r_ = 0 K	Constant value 0.48; *T*_c_ = 0.95*T*_m_	1.0	4.90 GPa
case 6	constant value 0.33; *T*_r_ = 293 K	constant value 0.48; *T*_c_ = 0.95*T*_m_	fitted to^48^; *T*_r_ = 0 K	Constant value 0.48; *T*_c_ = 0.95*T*_m_	1.7	6.81 GPa

The changed transition pressures at 293 K at some given shear stress levels are also listed.

It can be found from Fig. 5 that the phase boundary may move left or right of the hydrostatic line in the presence of shear stress, and its actual occurrence is sensitively correlated with the *σ*(*P*, *T*) relationships for both phases. As shown in Table 4, at 293 K, the transition pressure decreases to approximately 0.64 GPa in case 3, and increases to approximately 4.90 GPa in case 5 and 6.81 GPa in case 6. This variation range covers most scattered data measured in different experiments. Moreover, if the shear stress continues to increase, the transition pressure will display a greater change. Zirconium is known to be sensitive to various impurities, and a tiny amount of impurities, even in the order of ppm, may lead to great changes in its mechanical properties, such as hardness and yield strength. Thus, various samples in different experiments may satisfy various *σ*(*P*, *T*) relationships and hold different shear stresses. In addition, in static high-pressure experiments, powder samples are often used, as a result, shear stresses may be generated inside particles even under quasi-hydrostatic external loading conditions due to the friction between sample particles. Particularly, if fine powder samples are used, since the abundant particle surfaces act as obstacles to dislocation movement, the strain-hardening inside particles may be very strong, resulting in the emerge of high shear stresses inside particles. In^50^, it was found that before and after α-ω transformation, the maximum deviatoric stress in pure zirconium rapidly changed from 0.18 GPa to 1.18 GPa over a pressure interval about 1.0~1.3 GPa, which started at about 5.3 GPa, a pressure very close to the transition pressure 6.0 GPa. Consequently, the deviatoric stress at the transition pressure can be estimated to be about 0.72~0.88 GPa by linear interpolation. However, our most examinations were performed under the shear stress state J_2_ = 1 GPa^2^. Assuming that the shear stresses in the three principal directions in stress space are the same, the corresponding shear strength is 0.84 GPa. They are consistent. Therefore, the *σ*(*P*, *T*) relationships and the shear stress levels assumed in the six situations are valid in actual experiments. This means that the results predicted though our calculations are reasonable, thus it is understandable that the large scatter of the experimental measured transition pressure of the α-ω phase transformation of zirconium might be attributed to the difference in shear stress in different experiments.

As aforementioned in the introduction section, great controversial data on the ω-β transformation have been obtained in various experiments. One of the representatives is that both positive^22^ and negative^9,10^ slopes were reported for d*T*/d*P* in the vicinity of the α-ω-β triple-point. We believe that this controversy must be produced by some intrinsic factors instead of some uncontrolled errors in experiments, and among the factors, shear stress should be firstly considered. Our mEOS agrees with the phase diagram with a positive slope of d*T*/d*P* at the α-ω-β triple point; hence, it is critical to ascertain whether the slope would become negative in the presence of shear stress. In other aspects, our theoretical hydrostatic phase diagram predicts that the pressure-induced ω-to-β transformation at room temperature occurs at approximately 56 GPa, much higher than the experimental value 28–38 GPa^6–8^. The shock-induced transformation is predicted to occur at approximately 37 GPa, which is also higher than the experimental value of approximately 26 GPa^7,23^. The preceding part of this article is devoted to explain these controversies.

**Figure 6:**
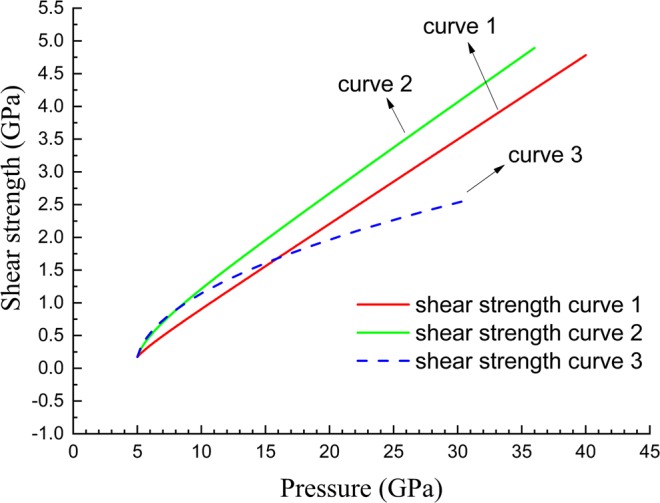
Shear strength curves constructed for estimating the movements of the ω-β phase boundary caused by shear stresses.

In principle, to estimate the change of transition pressure caused by shear stress, the shear stress states should be given in advance. However, it is difficult to re-identify the actual shear stress states in those experiments performed to measure the phase boundary. Generally, if the temperature is far away from the melting point, the shear stress will monotonically increase with the increasing hydrostatic pressure. Therefore, we artificially constructed three appropriate *J*_2_–P relationships to depict the shear stress variation with the increasing experimental pressure. Figure 6 shows the corresponding shear strength calculated according to the classical Mises yield model.

**Figure 7:**
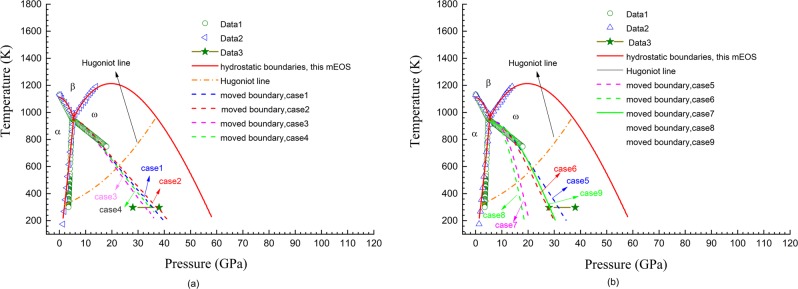
Variations of the ω-β phase diagram of zirconium caused by shear stresses. The results are examined under the conditions listed in Table 5. Data 1: from^9^; Data 2: from^22^; Data 3: from^6–8^.

Nine situations were examined for the ω-β transformation. The detailed *σ*(*P*, *T*) relationships and shear strength curves used in calculation are presented in Table 5, and the results are shown in Fig. 7. In case 1, for the ω-phase, *σ*_r_(*P*, *T*_r_) was assumed to be constant, with an experimental value of 0.29, and with *T*_r_ = 293.0 K, and *σ*_c_(*P*, *T*_c_(*P*)) was also assumed to be constant with the value 0.48 and with *T*_c_(*P*) = 0.95*T*_m_(*P*); for β-phase, *σ*_r_(*P*, *T*_r_) was fitted to the theoretical data obtained via first-principles calculations by Zhang *et al*.^48^ with *T*_r_ = 0 K, and *σ*_c_(*P*, *T*_c_(*P*)) was assumed to be constant with the value 0.45, and also *T*_c_(*P*) = 0.95*T*_m_(*P*). As shown by line-case1 in Fig. 7(a), when the shear strength varies with pressure along curve 1 shown in Fig. 6, the boundary becomes consistent with those experimental data obtained by Zhang *et al*.^9,10^, which has a negative slope of d*T*/d*P*. Moreover, the transition pressure at room temperature in this case becomes approximately 36 GPa, which agrees with the experimental measurements^6–8^. Similar results were obtained in cases 2–6, in which different *σ*(*P*, *T*) relationships constructed with various experimental and theoretic data in selected studies were used. In cases 7 and 8, the moved boundaries calculated by ZLC formalism become even lower than the experimental line obtained by Zhang *et al*.^9,10^. However, as shown by line-case9 in Fig. 7(b), if a lower level of shear stress represented by curve 3 in Fig. 6 is selected, the boundary moves back and coincides with the experimental data in^9,10^. It is simultaneously exhibited in Fig. 7 that the hydrodynamic Hugoniot line within ω-phase intersects with those altered ω and β phase boundaries at around 22 GPa. Taking the effects of the sample’s elastic-plastic behaviour on Hugoniot lines into consideration (a Hugoniot line will move close to the pressure axis in P-T space when taking the effects of sample’s elastic-plastic behaviour into consideration, hence the transition pressure should become a little higher than 22 GPa), it explains the experimental measurements that the shock-induced ω-β transformation occurs at around 26 GPa^7,23^.

**Table 5:** Poisson ratio variation relationships, i.e., *σ*(*P*, *T*) and shear strength curves that were used in the investigation of the ω-β phase boundary movements caused by shear stresses.

	ω-Zr		β-Zr		Shear strength curve
	*ν*_r_(*P*, *T*_r_)	*ν*_c_(*P*, *T*_c_)	*ν*_r_(*P*, *T*_r_)	*ν*_c_(*P*, *T*_c_)	
case 1	constant value 0.29; *T*_r_ = 293 K	Constant value 0.48; *T*_c_ = 0.95*T*_m_	fitted to^48^; *T*_r_ = 0 K	constant value 0.45; *T*_c_ = 0.95*T*_m_	curve 1
case 2	constant value 0.31; *T*_r_ = 293 K	Constant value 0.48; *T*_c_ = 0.95*T*_m_	fitted to^48^; *T*_r_ = 0 K	constant value 0.45; *T*_c_ = 0.95*T*_m_	curve 1
case 3	constant value 0.29; *T*_r_ = 293 K	Constant value 0.48; *T*_c_ = 0.95*T*_m_	fitted to^49^; *T*_r_ = 0 K	constant value 0.45; *T*_c_ = 0.95*T*_m_	curve 2
case 4	constant value 0.31; *T*_r_ = 293 K	Constant value 0.48; *T*_c_ = 0.95*T*_m_	fitted to^49^; *T*_r_ = 0 K	constant value 0.45; *T*_c_ = 0.95*T*_m_	curve 2
case 5	fitted to^47^; *T*_r_ = 293 K	Constant value 0.48; *T*_c_ = 0.95*T*_m_	fitted to^48^; *T*_r_ = 0 K	constant value 0.45; *T*_c_ = 0.95*T*_m_	curve 1
case 6	fitted to^47^; *T*_r_ = 293 K	Constant value 0.48; *T*_c_ = 0.95*T*_m_	fitted to^49^; *T*_r_ = 0 K	constant value 0.45; *T*_c_ = 0.95*T*_m_	curve 2
case 7	fitted to^48^; *T*_r_ = 0 K	Constant value 0.48; *T*_c_ = 0.95*T*_m_	fitted to^48^; *T*_r_ = 0 K	constant value 0.45; *T*_c_ = 0.95*T*_m_	curve 1
case 8	fitted to^48^; *T*_r_ = 0 K	Constant value 0.48; *T*_c_ = 0.95*T*_m_	fitted to^49^; *T*_r_ = 0 K	constant value 0.45; *T*_c_ = 0.95*T*_m_	curve 2
case 9	fitted to^48^; *T*_r_ = 0 K	Constant value 0.48; *T*_c_ = 0.95*T*_m_	fitted to^49^; *T*_r_ = 0 K	constant value 0.45; *T*_c_ = 0.95*T*_m_	curve 3

For shear strength curve, please refer to Fig. 6.

The shear stress levels represented by the three curves in Fig. 6 are not high, especially along curve 3. The shear strength does not exceed 2.5 GPa, even when the pressure rises to 30.0 GPa. Thus, based on the quantitative investigations in this work, it is reasonable to believe that the controversies on the ω-β transformation of zirconium are mainly induced by the inevitable shear stresses in the experiments.

## Summary and Conclusion

Significant debate has been noted in previous experiments for the α-ω and ω-β phase transformations in zirconium. The initial pressure of the α-to-ω transformation at room temperature was reported to spread in a range from 0.25 to 7.0 GPa^1–5,11,12^, while the hydrostatic transition pressure was believed to be approximately 2.2 GPa^4–7^. Earlier measurements of the ω-β phase boundary exhibited a positive d*T*/d*P* slope near the α-ω-β triple-point^22^, whereas recent experiments performed by Zhang *et al*.^9,10^ revealed a negative slope even at the α-ω-β triple-point. The ω-to-β transformation pressure at room temperature was also found to be controversial, varying from 28 to 38 GPa^6–8^. Similar controversies were simultaneously discovered in other extensive substances. How to explain these controversies, especially in a quantitative way, remains an open issue. In this article, we present a successful quantitative explanation for the controversies regarding the α-ω and ω-β transformations in zirconium by applying a nonhydrostatic thermodynamic formalism we proposed. We believe that the explanation is also appropriate for similar controversies observed in other substances.

## Data Availability

All Data generated or analysed during this study are included in the article.
